# Efficacy and safety of adult human bone marrow-derived, cultured, pooled, allogeneic mesenchymal stromal cells (Stempeucel®): preclinical and clinical trial in osteoarthritis of the knee joint

**DOI:** 10.1186/s13075-016-1195-7

**Published:** 2016-12-20

**Authors:** Pawan Kumar Gupta, Anoop Chullikana, Mathiyazhagan Rengasamy, Naresh Shetty, Vivek Pandey, Vikas Agarwal, Shrikant Yeshwant Wagh, Prasanth Kulapurathu Vellotare, Devi Damodaran, Pachaiyappan Viswanathan, Charan Thej, Sudha Balasubramanian, Anish Sen Majumdar

**Affiliations:** 1 Stempeutics Research Pvt Ltd, Akshay Tech Park, No. 72 & 73, 2nd Floor, EPIP Zone, Phase I-Area, Whitefield, Bangalore, 560066 India; 2 M.S Ramaiah Medical College & Hospitals, MSR Nagar, MSRIT Post, Bangalore, 560054 India; 3 Kasturba Medical College and Hospital, Madhav Nagar, Manipal 576104 India; 4 Sanjay Gandhi Post Graduate Institute of Medical Sciences, Raebareli Road, Lucknow, 226014 India; 5 Jehangir Clinical Development Center, Jehangir Hospital, 33, Sasoon Road, Pune, 411001 India; 6 Manipal University, Manipal, India

**Keywords:** Mesenchymal stromal cells, Osteoarthritis, Cell therapy

## Abstract

**Background:**

Osteoarthritis (OA) is a common and debilitating chronic degenerative disease of the joints. Currently, cell-based therapy is being explored to address the repair of damaged articular cartilage in the knee joint.

**Methods:**

The in vitro differentiation potential of adult human bone marrow-derived, cultured, pooled, allogeneic mesenchymal stromal cells (Stempeucel®) was determined by differentiating the cells toward the chondrogenic lineage and quantifying sulfated glycosaminoglycan (sGAG). The mono-iodoacetate (MIA)-induced preclinical model of OA has been used to demonstrate pain reduction and cartilage formation. In the clinical study, 60 OA patients were randomized to receive different doses of cells (25, 50, 75, or 150 million cells) or placebo. Stempeucel® was administered by intra-articular (IA) injection into the knee joint, followed by 2 ml hyaluronic acid (20 mg). Subjective evaluations—visual analog scale (VAS) for pain, intermittent and constant osteoarthritis pain (ICOAP), and Western Ontario and McMaster Universities Osteoarthritis (WOMAC-OA) index—were performed at baseline and at 1, 3, 6, and 12 months of follow-up. Magnetic resonance imaging of the knee was performed at baseline, and at 6 and 12 months follow-up for cartilage evaluation.

**Results:**

Stempeucel® differentiated into the chondrogenic lineage in vitro with downregulation of Sox9 and upregulation of Col2A genes. Furthermore, Stempeucel® differentiated into chondrocytes and synthesized a significant amount of sGAG (30 ± 1.8 μg/μg GAG/DNA). In the preclinical model of OA, Stempeucel® reduced pain significantly and also repaired damaged articular cartilage in rats. In the clinical study, IA administration of Stempeucel® was safe, and a trend towards improvement was seen in the 25-million-cell dose group in all subjective parameters (VAS, ICOAP, andWOMAC-OA scores), although this was not statistically significant when compared to placebo. Adverse events were predominant in the higher dose groups (50, 75, and 150 million cells). Knee pain and swelling were the most common adverse events. The whole-organ magnetic resonance imaging score of the knee did not reveal any difference from baseline and the placebo group.

**Conclusion:**

Intra-articular administration of Stempeucel® is safe. A twenty-five-million-cell dose may be the most effective among the doses tested for pain reduction. Clinical studies with a larger patient population are required to demonstrate a robust therapeutic efficacy of Stempeucel® in OA.

**Trial registration:**

Clinicaltrials.gov NCT01453738. Registered 13 October 2011.

## Background

Osteoarthritis (OA) is a common and debilitating chronic degenerative disease of large joints, especially the hip and knee, characterized by a loss of articular cartilage, subchondral sclerosis, and marginal osteophyte formation. Worldwide, approximately 9.6% of men and 18% of women aged ≥60 years have symptomatic osteoarthritis [1]. Current treatment in early-stage OA includes weight reduction, quadriceps strengthening exercises, non-steroidal anti-inflammatory drugs, intra-articular (IA) glucocorticoid injections, viscosupplements, and bracing [2–4]. Total joint arthroplasty is the mainstay treatment for end-stage OA of the knee joint, which is often associated with serious and life-threatening complications including increase risk of infection [5].

Currently, cell therapy- and tissue engineering-based approaches are being used to address the issue of repair of damaged articular cartilage. This includes autologous cultured chondrocytes and mesenchymal stromal cells (MSCs) obtained from various tissues that are used for transplantation into the cartilage lesion. Autologous chondrocyte implantation has inherent disadvantages such as a two-stage surgical procedure (harvesting healthy cartilage and transplanting culture-expanded chondrocytes from that sample) that may cause further cartilage damage and degeneration [6, 7], and chondrocyte dedifferentiation during culture that might result in fibrocartilage rather than hyaline cartilage formation [6, 8]. Thus, autologous or allogeneic MSCs are rapidly emerging as an investigational product for cartilage repair [9–11]. The anti-inflammatory and immunomodulatory properties of MSCs suggest that these cells can reduce inflammation and pain reduction in the knee. Concurrently, MSCs may initiate the repair process of the damaged cartilage by differentiating into chondrocytes, as well as by inducing proliferation and maturation of the remaining healthy chondrocytes or by inducing differentiation of chondroprogenitors [12]. A whole host of growth factors, biological modulators, and extracellular matrix proteins produced by MSCs may play a pivotal role in enhancing neocartilage formation [12].

Several preclinical studies and clinical trials have been conducted using MSCs which have reported the safety and therapeutic effect of its administration in patients with OA, although the majority of these studies have been conducted as single-dose, single-arm pilot studies [13–15]. Hence, there is a need for randomized, double-blind, controlled clinical trials. We have carried out in vitro studies to show the differentiation efficiency of adult human bone marrow-derived, cultured, pooled, allogeneic mesenchymal stromal cells (Stempeucel®) into the chondrogenic lineage and the expression of chondrocyte-specific markers. In order to determine if Stempeucel® is efficacious in a preclinical model, we have administered these cells intra-articularly into the knee joints of rats with mono-iodoacetate (MIA)-induced OA. After completion of these studies, a phase 2 dose-finding clinical study was initiated to evaluate the safety (primary endpoint), potential efficacy, and appropriate dose (secondary endpoints) of IA administration of Stempeucel® in patients with OA of the knee joint.

## Methods

### Production and characterization of Stempeucel® and placebo

Stempeucel® is a bone marrow-derived, ex vivo expanded, pooled, allogeneic human MSC population that has been characterized previously [16, 17]. The pooled cells were manufactured in an approved Good Manufacturing Practice (GMP) facility from bone marrow-derived MSCs (BMMSCs) of three different healthy volunteers to produce a working cell bank (WCB). The pooled MSCs from the WCB were further expanded to manufacture the investigational medicinal product, Stempeucel®. The cells expressed all markers characteristic of MSC, were negative for hematopoietic surface antigens, and also efficiently differentiated into osteocytes, chondrocytes, and adipocytes in vitro [16]. Two hundred million expanded BMMSCs were cryopreserved and stored in 15 ml PLASMA-LYTE A (Baxter, Deerfield, Illinois) containing 5% human serum albumin and 10% dimethyl sulfoxide (DMSO) in cryobags (MacoPharma, Mouvaux, France). Placebo contained 15 ml PLASMA-LYTE A in similar cryobags. The investigational medicinal product (IMP) specification is given in Table 1.

**Table 1:** Investigational medicinal product (IMP) specification

S. no	Description	Specifications	
1	Morphology	Cells are fibroblastic and spindle-shaped in active growing conditions Cells are intact and round in shape after trypsin action	
2.	Cell count	180 to 220 million cells per bag	
3.	Viability	≥85%	
4.	Cell phenotype	CD 73 >80% CD105 > 80% CD 90 > 80% CD 166 > 80%	CD 34 <5% CD 45 < 5% CD 133 < 5% CD 14 < 5% CD19 < 5% HLA-DR < 5%
5.	Karyotyping	Normal, 46 XY	
6.	Mycoplasma PCR ELISA	Not detected	
7.	Sterility test	Must comply	
8.	Differentiation assay to adiopocyte, osteocyte, and chondrocyte	Confirmation of differentiation	

### In vitro studies show differentiation of Stempeucel® to the chondrogenic lineage and quantification of sulfated glycosaminoglycan

The chondrogenic differentiation potential of six Stempeucel® batches was evaluated in monolayer cultures using chondrogenesis induction medium (catalog no. A10071-01; Gibco). Cells were plated at a density of 1000 cells/cm^2^ in six-well plates and cultured in DMEM-KO with 10% fetal bovine serum (FBS) and 2 ng/mL basic fibroblast growth factor (bFGF) until 80% confluency. The cells were induced to differentiation in chondrogenic induction medium (Gibco) for 21 days; the medium was replenished every 3 days and un-induced cells were harvested at 80% confluency and served as the corresponding control for background estimation. After 21 days of differentiation, the cells were trypsinized and pelleted by centrifugation at 1000 rpm and the chondrogenic differentiation was quantified by measuring the amount of sulfated glycosaminoglycan (sGAG) using a Blyscan kit (catalog no. B1000; Bicolor). The final sGAG content was represented after normalizing with total DNA content estimated using a Quant-iT Pico green kit (P7589; ThermoFisher, USA).

#### Quantitative RT-PCR for chondrogenic-specific gene *markers*

Total cellular RNA was isolated using an RNeasy mini kit (Qiagen) from the undifferentiated and the differentiated BMMSCs for chondroyte lineage (described above). The RNA samples were treated with RNAse free DNase I (Ambion), and reverse-transcribed into cDNA using a high-capacity cDNA reverse transcription kit (Applied Biosystems) according to the manufacturer’s instructions. Real-time PCR was carried out using the SYBR green kit (catalog no. 4309155; Applied Biosystems) using step one plus (Applied Biosystems). Beta actin served as the internal control. The sequences for the gene-specific primers are as follows: Sox9, forward TTTCCAAGACACAAACATGA, reverse AAAGTCCAGTTTCTCGTTGA; Col2A, forward TTTCCCAGGTCAAGATGGTC, reverse TCACCTGGTTTTCCACCTTC. Ct values were normalized to the housekeeping gene β-actin.

### Preclinical model of osteoarthritis

Ten-week-old male Wistar rats (*n* = 80) weighing between 175 and 285 g were used for the preclinical study. The experimental protocol was approved by the Institutional Animal Ethics Committee (IAEC), and the experiment was carried out at a CRO animal facility.

Bilateral osteoarthritis was induced in rats (*n* = 74) by injection of MIA (Sigma-Aldrich, USA) into the knee joints according to published methods [18–20]. Briefly, the animals were anesthetized with 3% isoflurane; 1 mg MIA dissolved in 50 μl saline was delivered into the articular cavity. The rats (*n* = 6) receiving only the saline solution served as sham control animals (group I) throughout the experiment. The pain sensitivity of the knee joints was measured before MIA injection and once every week after MIA injection as described by Di Cesare et al. [21]. The Pressure Application Measurement device (PAM; Ugo Basile, Italy) was used to measure the mechanical pain threshold of the knee joints. The gram-force (gf) that elicited the limb withdrawal was recorded. Three weeks after MIA injection, animals displaying PAM values that ranged between 272 and 601 gf were considered to have developed OA and were selected for the study (*n* = 60). Prior to performing the experiment described in this paper, we had conducted a validation study in which the range of PAM values was evaluated against gross pathology and histological evidence of OA in rats (data not shown). The range of PAM values was selected accordingly prior to randomizing the animals in the current study. Rats were divided into four different groups and each group consisted of 15 rats (Table 2). Sham control animals (group I) showed an average PAM value of 870 ± 138 gf.

**Table 2:** Animal grouping and sacrifice schedule

Group		Animals sacrificed (no of rats)*		
		4 weeks	8 weeks	12 weeks
Group 1	Sham control	2	2	2
Group 2	Vehicle control	3	6	6
Group 3	Hyaluronic acid (HA)	3	6	6
Group 4	Stempeucel® low dose + HA (6 × 10^5^ cells/joint)	3	6	6
Group 5	Stempeucel® high dose + HA (1.3 × 10^6^ cells/joint)	3	6	6

*Number of animals sacrificed at each time point after Stempeucel® administration

#### Stempeucel® administration in MIA-induced rats

At day 0, animals in both groups 1 (sham control) and group 2 (vehicle control) received 60 μl vehicle (Plasmalyte A; Baxter) through the IA route delivered by a 27-gauge needle. For group 3 animals, 30 μl hyaluronic acid (HA; Hyalgan®; Fidia Pharmaceuticals, Italy) was injected followed by 30 μl vehicle. For group 4 and 5 rats, freshly-thawed Stempeucel® was used after washing in Plasmalyte A to remove DMSO and resuspending in Plasmalyte A. Stempeucel® was administered at two different doses: 6 × 10^5^ cells/joint (low dose; human equivalent dose (HED) of 25 million cells) and 1.3 × 10^6^ cells/joint (high dose; HED of 50 million cells). Both doses were formulated in 30 μl Plasmalyte A and injected into group 4 (low-dose group) and group 5 (high-dose group) animals, respectively. Cell administration was immediately followed by 30 μl HA injection. In order to reduce the xenogeneic rejection of cells, cyclosporine A (CsA; Novartis, Switzerland) was injected subcutaneously into all the experimental animals at a dose of 10 mg/kg daily for an initial 1 week starting at day –3 of cell injection, after which daily CsA administration was continued orally at the same dose until the end of the study. The pain response was measured at weekly intervals up to week 10. The final measurements were taken at week 12.

#### Gross and histological evaluation of cartilage repair in preclinical model

The treatment regimen and the number of animals sacrificed after BMMSC administration at various time points are shown in Table 2. Rats from all the groups were sacrificed at 4, 8, and 12 weeks after vehicle/HA/Stempeucel® administration. The knee joints were dissected and the cartilage surface was visualized macroscopically on the exposed joints, and the distal femur from the right joints was dissected and processed for histological analysis.

All joint specimens were fixed in 10% formalin buffer and then decalcified in 10% EDTA (RFCL, India) for 2 weeks; the decalcification solution was changed twice in a week. The joints were embedded in paraffin; serial sagittal sections (5-μm thick) were prepared and stained with hematoxylin and eosin (H&E) and Safranin-O fast green staining for proteoglycan visualization and estimation [22]. The severity of articular damage was evaluated using the Osteoarthritis Research Society International (OARSI) grading system [23, 24] on the H&E-stained sections. The binding intensity of Safranin-O to the sGAG was quantified using imageJ software [25]. The region of interest (ROI) around the cartilage area (*n* = 3 per section) was selected, and the intensity of red (R), green (G), and blue (B) were measured and the proportion of red staining was calculated using the equation r = R/ (R^2^ + G^2^ + B^2^)^1/2^ where, R is the intensity of red and 'r' refers to the intensity of the red fraction with respect to other primary colors [26]. The sections were graded and quantified by an independent observer blinded to the treatment groups.

### Clinical study design and enrollment criteria

**Fig. 1:**
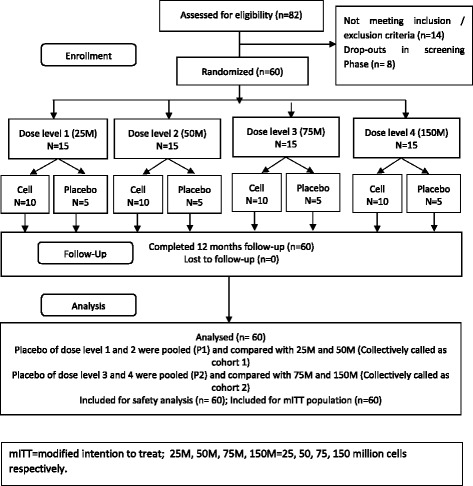
CONSORT flow chart showing the number of patients randomized, followed-up, and analyzed. *M* million cells, *Cell* Stempeucel®, *mITT* modified intention to treat

This was a randomized, double-blind, multicentric, placebo-controlled, phase II study assessing the safety and efficacy of IA Stempeucel® in patients with OA of knee. The study was conducted in accordance with the Good Clinical Practice (GCP) guidelines as issued by the International Conference on Harmonization (ICH/135/95, July 2002), Schedule Y of the Drugs and Cosmetics Rules, 1945, Ethical guidelines for biomedical research on human participants prepared by the Indian Council of Medical Research in 2006 and the Declaration of Helsinki (64th WMA General Assembly, Fortaleza, Brazil, October 2013). Approval was obtained from the Central Drugs Standard Control Organization (Indian FDA) and the institutional ethics committees of the five participating hospitals. The study was registered in the National Institute of Health registry of clinical trials (https://clinicaltrials.gov/ct2/show/NCT01453738). An independent data safety monitoring board was formed comprising of drug safety physicians and an expert in the therapeutic area to monitor the safety data at predefined intervals during the progress of the study. The study was conducted from November 2011 to November 2013. Written informed consent was obtained from all participants before screening. Of the 82 patients screened, 62 patients were randomized to the study from five centers. Two patients dropped out from the study after randomization but before IMP administration; thus 60 patients received the IMP. Four dose levels were studied in this trial: 25, 50, 75, and 150 million cells (25 M, 50 M, 75 M, and 150 M, respectively). At each dose level, 15 patients were randomized into two groups (Stempeucel® and placebo) in a 2:1 ratio using a computer generated randomization. Thus, 10 subjects received Stempeucel® and 5 subjects received placebo at each dose level (Fig. 1). As this was the first study of IA administration of Stempeucel® in OA patients, no formal sample size calculation was performed. Eligibility criteria of the patients in the trial are given in Table 3.

**Table 3:** Subject eligibility criteria

Inclusion criteria 1. Males or females in the age range 40–70 years (both inclusive) 2. Radiographic evidence of grade 2 to 3 osteoarthritis (OA) based on the Kellgren and Lawrence radiographic entry criteria* 3. History of primary idiopathic OA of the knee characterized by pain which required intake of analgesics 4. Self-reported difficulty in at least one of the following activities attributed to knee pain: lifting and carrying groceries, walking 400 meters, getting in and out of a chair, or going up and down stairs 5. Patients who had been on stable medication, including non-steroidal anti-inflammatory drugs (NSAIDs)/opioid or opiate analgesics, for the past 3 months 6. Female patients of childbearing age who agreed to use accepted methods of contraception during the course of the study 7. Ability to provide written informed consent
Exclusion criteria 1. Prior or ongoing medical conditions (e.g., concomitant illness, psychiatric condition, alcoholism, drug abuse), medical history, physical findings, electrocardiogram (ECG) findings, or laboratory abnormality that, in the investigator’s opinion, could adversely affect the safety of the subject, makes it unlikely that the course of treatment or follow-up would be completed or could impair the assessment of study results 2. History of surgery or major trauma to the study joint 3. Arthroscopy on the study joint in the previous 12 months 4. Signs of active study joint inflammation including redness, warmth, and/or, if qualifying with OA of the knee, a large, bulging effusion of the study knee joint with the loss of normal contour of the joint at the screening visit or at the baseline examination 5. Patients who received intra-articular steroids or hyaluronan within the last 3 months 6. Infections in or around the knee 7. Patients awaiting a replacement knee or hip joint 8. Patients with other conditions that caused pain 9. Patients with deformity of the knee joint 10. Significantly incapacitated or disabled and would be categorized as ACR Functional Class IV (largely or wholly incapacitated) or unable to walk without assistive devices 11. Patients with other known rheumatic or inflammatory disease such as rheumatoid arthritis 12. Other pathologic lesions on X-rays of knee 13. Positive hepatitis B surface antigen, hepatitis C antibody test, anti-HIV antibody test, or Rapid Plasma Reagin (RPR) 14. History of bleeding disorders 15. Known hypersensitivity to hyaluronan or animal sera 16. For women of child-bearing potential: positive pregnancy test or lactating (females who were planning pregnancy within the next year were excluded)

*If both knees of a patient were eligible to be included in the study, the knee to be included was as per investigator judgment

### Preparation of IMP at the clinical trial sites

Block randomization (block size 5) was performed centrally by a biostatistician using PROC PLAN in SAS. The IMP (Stempeucel® or placebo) was shipped in a cryoshipper (temperature –185 to –196 °C) to clinical trial sites whenever each patient was eligible for the study. Preparation of IMP for injection was performed under a validated biosafety cabinet by a trained person independent of the investigator’s team. A cryobag containing IMP was thawed at 37 °C in a water bath. The cell suspension was diluted to 100 ml using PLASMA-LYTE A using two 50-ml centrifuge tubes. The cell pellet was resuspended in 2 ml PLASMA-LYTE A (for the 25- and 50-million-cell dose groups) and in 4 ml PLASMA-LYTE A (for the 75- and 150-million-cell dose groups) based on the viable cell count and was loaded to a blinded syringe. For the placebo preparation, a similar amount of PLASMA-LYTE A was loaded to a blinded syringe. The IMP (Stempeucel® or placebo) was presented to the investigators in a blinded syringe (using semitransparent tape) in a temperature-controlled transport box at 2–8 °C. It was not possible to distinguish between Stempeucel® and placebo upon visual inspection of the blinded syringes.

### Injection protocol for the clinical study

Pre-medication (hydrocortisone 100 mg IV and pheniramine maleate 45.5 mg IV) was administered 15–30 min before administration of the IMP to prevent the possibility of a potential anaphylactic reaction to the allogeneic cells. The IA injections were performed by qualified and experienced investigators (either orthopedician or rheumatologist) using a 2.0-inch (5.1-cm) 20-gauge needle as a lateral midpatellar injection (an injection into the patellofemoral joint). IMP injection was followed by injection of 2 ml hyaluronic acid (20 mg; Hyalgan, Fidia Farmaceutici S.p.A., Italy). Patients were hospitalized for the procedure, and were monitored for 24 h after the injection. Patients were discharged after inspection of the target joint, a general physical examination, and vital signs evaluation.

### Follow-up for the clinical trial patients

Patients were followed up at 1 week, and 1, 3, and 6 months after injection of IMP. The clinical data were unblinded after 6 months and patients were further followed-up for both safety and efficacy until 12 months after the injection. Safety assessments included monitoring of all adverse events (AEs), assessment of electrocardiogram (ECG) parameters, hematological (complete blood count including erythrocyte sedimentation rate) and biochemical (liver function tests, kidney function tests, and lipid profile) values, physical examination, and vital signs measurements. Adverse events were captured by interviewing the subjects and laboratory data evaluations during the visits. The efficacy endpoints included improvement in pain from OA graded on a visual analog scale (VAS) from 0 to 10 (0 = no pain; 10 = worst pain); also, the Western Ontario and McMaster Universities Osteoarthritis (WOMAC-OA) index (total score, pain, stiffness, and physical function scores) and intermittent and constant osteoarthritis pain (ICOAP) was used (constant and intermittent pain score) to evaluate pain and function of the joint. X-ray of the knee was carried out at baseline, and at 3 and 6 months follow-up. Magnetic resonance imaging (MRI) of the knee was carried out at baseline, and at 6 and 12 months follow-up. MRI of the knee was performed using a 1.5-T whole body scanner and a circumferential eight-channel knee coil. Proton density T2-weighted sequences were captured: sagittal intermediate, axial intermediate, and coronal intermediate for assessing osteophytes and cartilage (for scoring cartilage signal and morphology, marginal osteophytes, subarticular bone marrow abnormality, subarticular cysts, and subarticular bone attrition); sagittal PD FS for assessing menisci and cruciate ligaments (for scoring of ACL, PCL, medial, and lateral menisci); and sagittal 3D FIESTA-C for assessing cartilage surface and superficial erosions (for scoring cartilage signal and morphology). Images were scored to assess the whole-organ magnetic resonance imaging score (WORMS) by two experienced radiologists using combined reads. In case of non-concurrence, advice was taken from a third independent radiologist whose report was considered final. WORMS scoring used in this study was modified for calculation of total WORMS score. The parameters included were articular surface features which include cartilage signal and morphology, subarticular bone marrow abnormality, subarticular cysts, subarticular bone attrition, and marginal osteophytes. They were scored for all 14 compartments of the knee joint. The compartment totals were added to obtain the overall knee joint score [27].

### Statistical analysis

#### Preclinical data

GraphPad Prism software was used to calculate the statistical significance of all preclinical experimental data. The data are expressed as the mean ± SEM. Quantitative RT-PCR data analysis was performed using Student’s *t* test. Pain threshold differences between various treatment groups and the Safranin-O quantification were examined for statistical significance using two-way analysis of variance followed by a Bonferroni post-hoc test. The OARSI grade analysis was performed using one-way analysis followed by Kruskal–Wallis test. *P* < 0.05 denoted the presence of a significant difference between groups.

#### Clinical trial data

The SAS package (SAS® Institute Inc., USA, version 9.2) was used for statistical evaluation. For analysis purpose, subjects were grouped as follows: four treatment groups (25 M, 50 M, 75 M, and 150 M cell dose), and two placebo groups. Patients who received placebo in 25 M and 50 M dose levels were grouped into one placebo group (both received the IMP in 2 ml PLASMA-LYTE A; P1) and those who received placebo corresponding to the 75 M and 150 M dose groups (both received the IMP in 4 ml PLASMA-LYTE A) were grouped into another group (P2). Thus, there were four treatment groups and two placebo groups forming a total of six groups. The treatment groups 25 M and 50 M were compared with P1 (the three groups collectively called cohort 1) and treatment groups 75 M and 150 M were compared with P2 (the three groups collectively called cohort 2). AEs were coded by the Medical Dictionary for Regulatory Activities (MedDRA) primary system organ class (SOC) and preferred term (PT). AEs were summarized descriptively by total number of AE(s) and compared between the six study groups. Continuous variables are presented as mean ± SD. The data distribution was visually examined for normality before applying the statistical tool for analysis. Comparisons among groups were conducted using Kruskal–Wallis test, with alpha set at 0.05 for significance.

## Results

### Differentiation efficiency of Stempeucel® into chondrogenic lineage in vitro

**Fig. 2:**
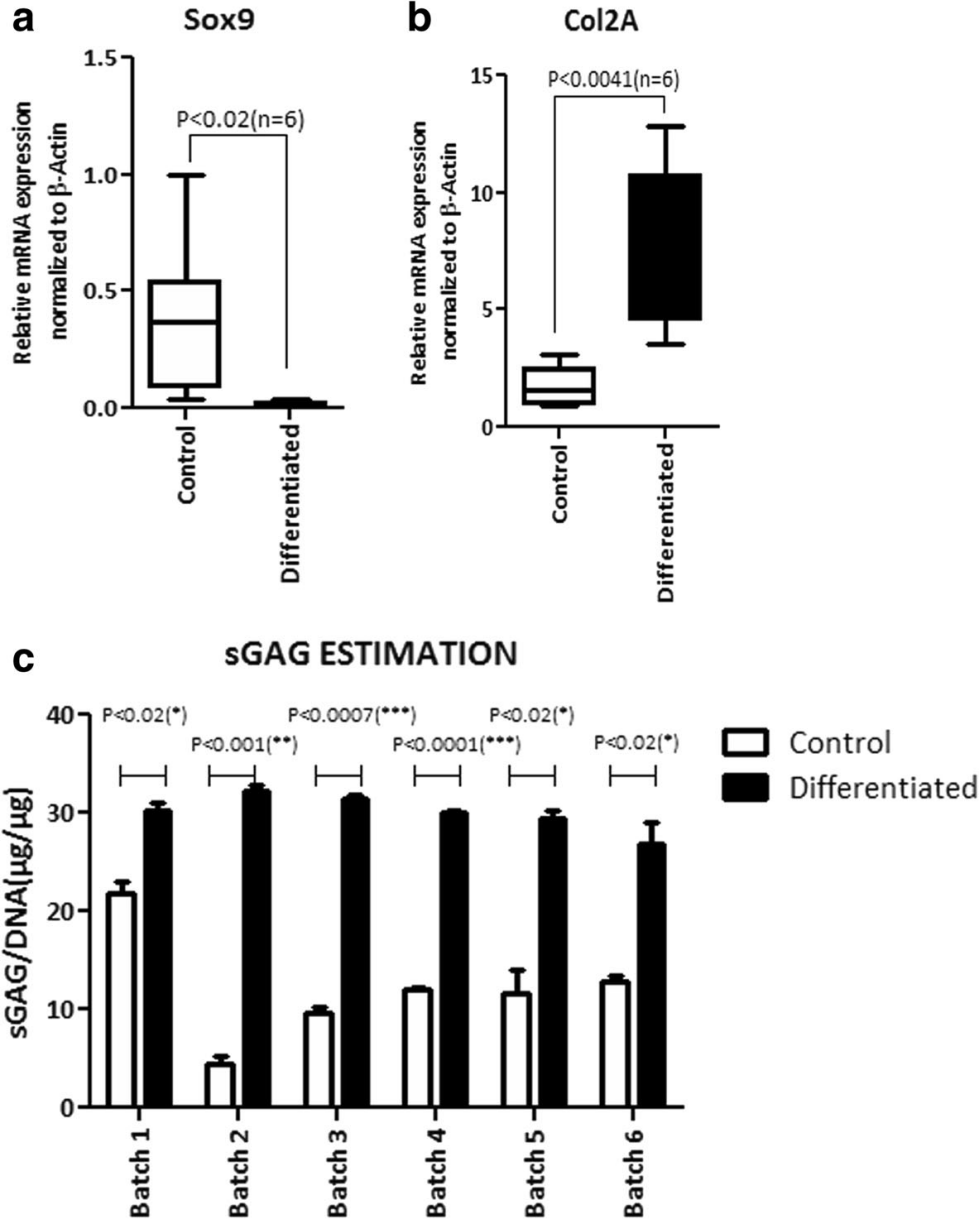
Quantification of gene expression and sGAG. Quantitative mRNA expression of **a** SOX9 and **b** Col2A in the control (*white bar*) and chondrogenically differentiated Stempeucel® (*black bar*) by real-time PCR analysis (*n* = 6). **c** Sulfated glycosaminoglycan (*sGAG*) content in the control (*white bar*) and chondrogenically differentiated Stempeucel® (*black bar*) by DMMB dye-binding assay. The sGAG values were normalized to the DNA content in the control and chondrogenically differentiated Stempeucel® (*n* = 6). Results are represented as mean with SEM

We evaluated the chondrogenic differentiation potential of Stempeucel® batches in monolayer cultures. The extent of differentiation was also assessed by mRNA expression of Sox9 and Col2A. We observed a significant downregulation of Sox9, which is an early inducer of chondrogenesis in the differentiated cells compared to the control (*P* < 0.02, *n* = 6), and upregulation of Col2A, the gene that encodes for type 2 collagen, a major cartilage matrix protein and a mature chondrocyte marker (Fig. 2a and b). Chondrogenic differentiation of BMMSCs was quantified by measuring the amalgamation of sGAG which is known to play a central role in cartilage homeostasis [28]. All six batches of Stempeucel® differentiated to chondrocytes and synthesized significant amount of sGAG (30 ± 1.8 μg/μg GAG/DNA) compared to that produced by the undifferentiated cells (12.07 ± 5.6 μg/μg GAG/DNA; *P* < 0.001, *n* = 6) (Fig. 2c). These data suggest that the pooled BMMSC samples efficiently differentiated into the chondrogenic lineage, confirming the presence of mature chondrocytes after differentiation.

### Intra-articular administration of Stempeucel® ameliorates OA-induced joint pain in a preclinical model

**Fig. 3:**
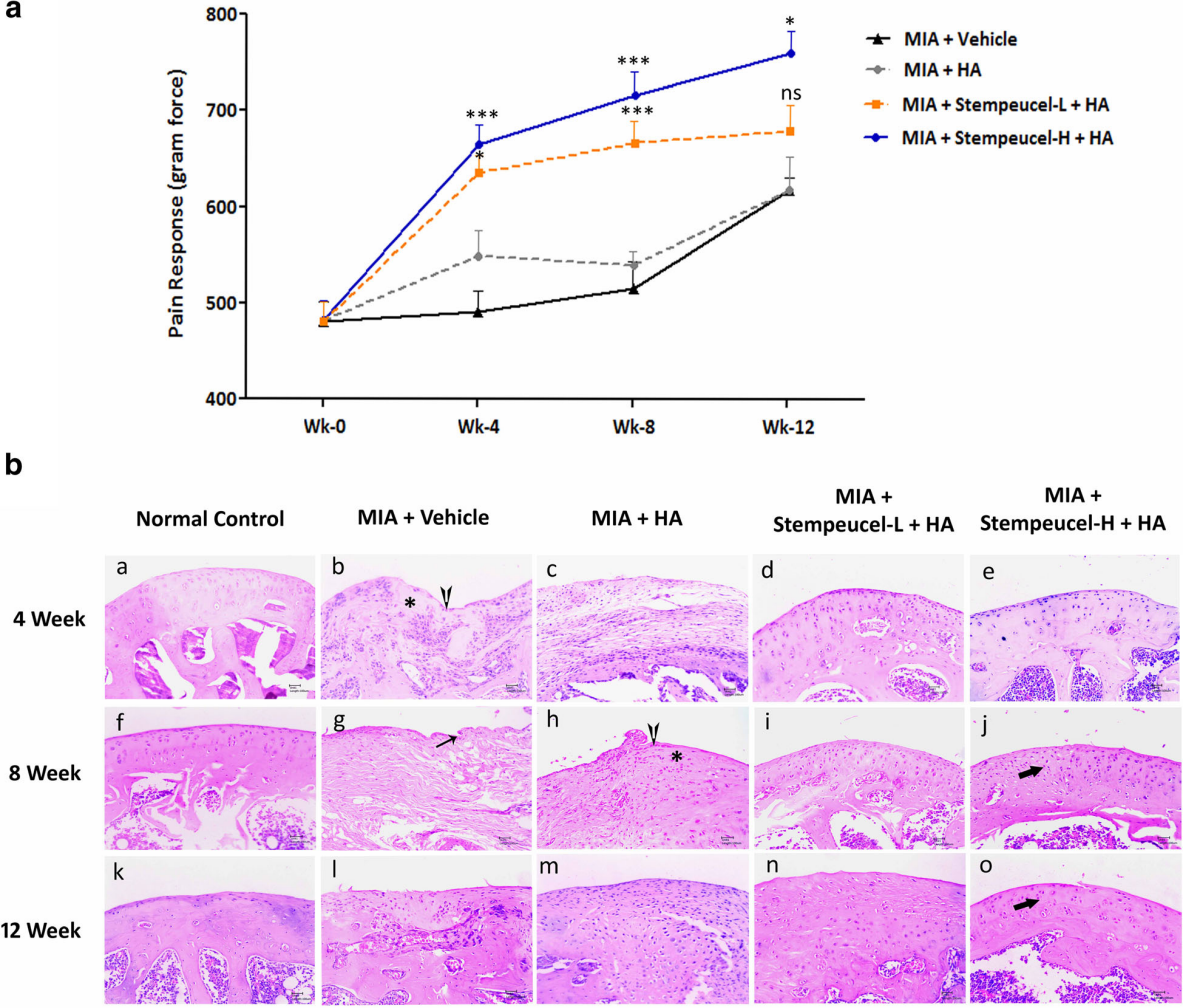
Effect of intra-articular injection of Stempeucel® on pain reduction and cartilage repair in an osteoarthritic rat model. **a** The effect of Stempeucel® on pain reduction at week 0 (before cell injection), and at weeks 4, 8, and 12 after cell injection. Data are presented as mean ± SEM. *P < 0.05, ***P < 0.001 versus hyaluronic acid (*HA*)-treated group. **b** Photomicrographs of representative joint sections of femoral condyle stained with H&E at 4 (a–e), 8 (f–j), and 12 weeks (k–o) after Stempeucel® treatment. Osteoarthritic changes, such as loss of chondrocytes (*), loss of cartilage (*vertical arrow*), and fibrillation (*thin arrow*) are evident in vehicle-treated and HA-treated joints. Proliferation of chondrocytes (*thick arrow*), regeneration, and repair of cartilage tissue was evident in Stempeucel®-treated groups. *Scale bars* = 100 μm, magnification 10×. *H* high dose of Stempeucel®, *L* Low dose of Stempeucel®, *MIA* mono-iodoacetate, *ns* not significant

The MIA-induced OA model was the first validated pain model of OA used to evaluate the analgesic and anti-inflammatory properties of therapeutic agents [20]. In our study, the mechanical pain threshold was measured weekly by PAM test. Pain threshold values from various time points are shown in Fig. 3a. Analysis of PAM scores indicates that animals treated with both low and high doses of Stempeucel® showed improvement in pain threshold from week 2 onwards as compared to vehicle or HA-treated animals and continued to improve until 8 weeks after cell injection. While the pain improvement appeared to reach saturation by 8 weeks with the low dose of cells, it continued to improve until 12 weeks in the animals that received a high dose of Stempeucel® (Fig. 3a). Animals treated with HA alone showed some improvement in pain threshold at the initial time period (week 3); however, this improvement was not found to be consistent. When comparing the PAM scores between the HA- and Stempeucel®-treated groups, animals treated with the high dose of cells exhibited a significant pain-reduction effect at week 4 (*P* < 0.001), week 8 (*P* < 0.001) and week 12 (*P* < 0.05), whereas the low-dose group showed a significant effect at week 4 (*P* < 0.05) and week 8 (*P* < 0.001), but not at week 12. These results clearly demonstrate that intra-articularly injected Stempeucel® is able to reduce the pain significantly in the MIA-induced OA rat model.

### Cartilage repair induced by Stempeucel® administration in a preclinical model

Macroscopic evaluation of articular cartilage was performed in rats at 4, 8, and 12 weeks after cell injection. Varying degrees of cartilage damage were observed in animals injected with MIA and subsequently administered with vehicle. These degenerative changes include cartilage fibrillation, erosion, and osteophyte formation (data not shown). The degree of cartilage damage was progressively reduced with both low and high doses of Stempeucel® + HA treatment, while only marginal change was noticed in a few HA-treated animals.

**Fig. 4:**
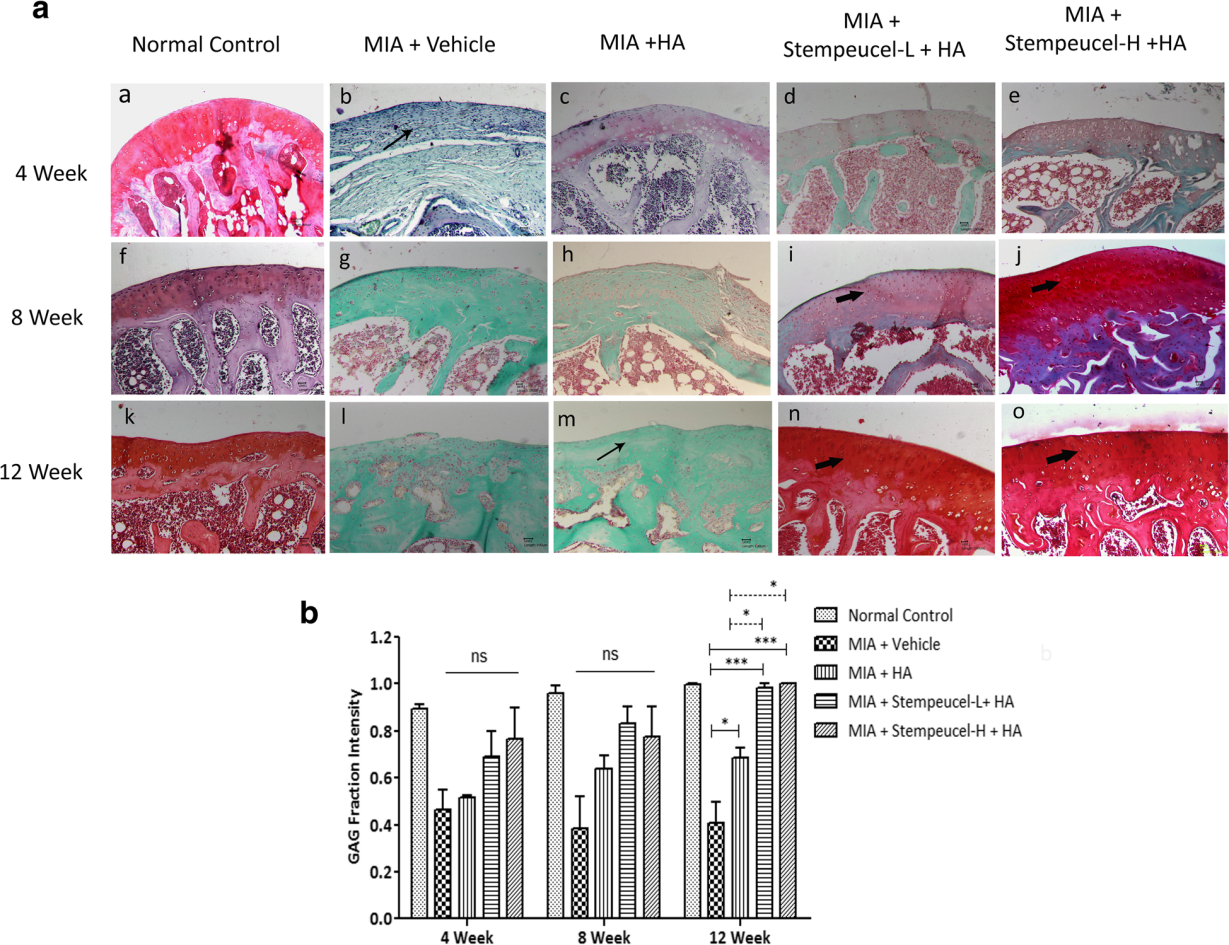
Histological evaluation of Safranin-O stained joint sections. **a** Photomicrographs of representative joint specimens of femoral condyle stained with Safranin-O at 4 (a–e), 8 (f–j), and 12 weeks (k–o) after Stempeucel® treatment. Loss of articular surface, roughening of cartilage and reduced staining of Safranin-O (*thin arrow*) were observed in vehicle- and HA-treated joints. Strongly stained Safranin-O-positive cartilage (*thick arrow*) with increased numbers of chondrocytes was seen in the Stempeucel®-treated groups. *Scale bars* = 100 μm, magnification 10×. **b** Sulfated glycosaminoglycan (*GAG*) fraction intensity was measured from histological images of Safranin-O-stained sections at weeks 4, 8, and 12. The intensity of Safranin-O staining is represented graphically, and the data are represented as mean ± SEM. At 12 weeks, the Stempeucel®-treated groups (both low (*L*) and high (*H*) dose) showed a significant improvement in the sGAG content compared to the disease control (mono-iodoacetate; *MIA*) and hyaluronic acid (*HA*)-treated groups. *P < 0.05, *** *P* < 0.001. *ns* not significant

Based on H&E staining of the cartilage tissue, we scored the femoral condyle section of rats using the OARSI grading system. As expected, sham control animals showed a normal histological appearance of the cartilage throughout the study (Fig. 3b, panels a, f, and k for weeks 4, 8, and 12, respectively). In the vehicle-treated animals, the OA joints showed fibrillation, and appreciable loss of chondrocytes was observed at both weeks 4 and 8 (Fig. 3b, panels b and g). By week 12, the cartilage damage was found to be severe and the hyaline tissue was replaced with fibrocartilage (Fig. 3b, panel l). The HA-treated animals showed marginal improvement in cartilage architecture (Fig. 3b, panels c, h, and m). In contrast, animals receiving both low and high doses of Stempeucel® showed almost intact cartilage with a larger number of chondrocytes (Fig. 3b, panels d, i, and n for a low dose of cells and panels e, j, and o for a high dose of cells). The calculated OARSI grades of the different treatment groups were compared at week 12. Although, both low- and high-cell doses showed histological improvement, a statistically significant reduction in OARSI grade was observed only in the high-dose Stempeucel®-treated animals (*P* < 0.05) in comparison to animals in the vehicle group. The Safranin-O stained area of the cartilage was greater in both the cell-treated groups (Fig. 4a, panels d, i, and n for the low dose and panels e, j, and o for the high dose). It is important to note that the intensity of Safranin-O staining increased progressively with both doses of Stempeucel®. In fact, at week 12, Safranin-O staining intensity was found to be comparable between the high- and low-cell dose animals and sham controls (Fig. 4a). In comparison to the HA- and vehicle-treated groups, sGAG intensity was found to be significantly higher in Stempeucel®-treated and in sham control animals at week 12 (Fig. 4b). These findings suggest that intra-articularly injected Stempeucel® + HA repaired MIA-induced articular cartilage damage in rats with OA of the knee joint.

### Clinical study

#### Demographics and baseline characteristics of patients

The demographics and other baseline characteristics of the enrolled patients are presented in Table 4. All six groups were mostly comparable in terms of baseline characteristics. Sixty patients were included in the modified intention to treat analysis (mITT) group. There was no premature unblinding of any patient. The patients’ age, sex, weight, height, and body mass index (BMI) were balanced across groups. High scores for VAS, WOMAC, and ICOAP suggest that these patients had severe pain and were balanced across all groups.

**Table 4:** Summary of Demographic Characteristics at Baseline

	Cohort 1				Cohort 2			
Parameter	25 M (*n* = 10)	50 M (*n* = 10)	P1 (*n* = 10)	*P* value	75 M (*n* = 10)	150 M (*n* = 10)	P2 (*n* = 10)	*P* value
Age (years)	58.10 ± 8.23	57.30 ± 9.45	54.90 ± 8.27	0.73	55.00 ± 6.72	54.00 ± 6.73	56.70 ± 5.19	0.6
Female (*n*)	7	8	10	NA	8	5	7	NA
Male (*n*)	3	2	0	NA	2	5	3	NA
Height (cm)	156.85 ± 9.64	157.30 ± 12.23	152.25 ± 9.72	0.39	158.40 ± 8.86	158.88 ± 9.30	159.70 ± 10.67	0.9
Weight (kg)	73.10 ± 15.86	69.00 ± 14.62	66.10 ± 7.67	0.45	71.30 ± 9.09	66.00 ± 9.13	66.90 ± 8.57	0.39
BMI (kg/m^2^)	29.73 ± 6.09	27.74 ± 4.16	28.84 ± 4.91	0.76	28.38 ± 2.38	26.33 ± 4.48	26.40 ± 3.99	0.3
WOMAC total	1315.8 ± 444.8	1498.4 ± 407.4	1239.6 ± 472.2	0.28	1470.6 ± 471.0	1388.1 ± 508.8	1382.0 ± 324.7	0.9
ICOAP total	45.7 ± 19.2	59.3 ± 21.7	49.3 ± 18.7	0.38	58.4 ± 20.7	46.4 ± 22.0	54.8 ± 17.8	0.54
VAS	60.9 ± 19.7	73.7 ± 15.2	61.0 ± 23.8	0.24	57.4 ± 29.0	46.6 ± 23.6	65.3 ± 12.2	0.11
WORMS total score	67.0 ± 19.8	78.8 ± 40.9	76.5 ± 23.5	0.65	71.3 ± 21.4	62.0 ± 17.9	70.8 ± 14.7	0.48
Kellgren and Lawrence Grade 2 (*n*)	4	1	3	NA	1	3	2	NA
Kellgren and Lawrence Grade 3 (*n*)	6	9	7	NA	9	7	8	NA

Values are shown as mean ± SD unless indicated otherwise

25 M, 50 M, 75 M, 150 M = 25, 50, 75, and 150 million cells, respectively

P1, P2 = placebo 1 and placebo 2, respectively

BMI body mass index, ICOAP intermittent and constant osteoarthritis pain, NA statistical comparisons for these groups have not been conducted due to too few samples, VAS visual analog scale, WOMAC Western Ontario and McMaster Universities

#### Procedural safety

All patients tolerated the procedure well in cohort 1 (25 M, 50 M, and P1) and cohort 2 (75 M, 150 M, and P2). Ten patients (1 in 50 M, 6 in 75 M, and 3 in 150 M dose groups) experienced pain and swelling at the injection site. The events were mild to moderate in severity, assessed as related to the IMP, and recovered upon conservative therapy. Among the 10 patients, one subject (150 M dose group) had IMP-related synovial effusion requiring hospitalization for one additional day of observation, thus meeting the criteria of a serious adverse event (SAE). Other SAEs in the study were determined to be unrelated to the IMP and were as follows: hysterectomy for menorrhagia in one subject in the 25 M group, suture-related complication and varicose vein in one subject in the P1 group, and hemorrhoidal hemorrhage and umbilical hernia, respectively, in one subject each in the P2 group.

#### Overall evaluation of adverse events

A total of 97 AEs were reported in 40 subjects (Table 5). The distribution of AEs in the different dose groups was as follows: 24 (25 M), 13 (50 M), 21 (P1), 17 (75 M), 11 (150 M), and 11 (P2). No patient died or was withdrawn from the study due to an AE. Most of the AEs were mild to moderate in severity. One severe AE was reported in each of 25 M (dyslipidemia), 50 M (anemia), 150 M (muscle hemorrhage), and P2 (umbilical hernia) groups. Physical examination and vital signs data were unremarkable after the injection. In cohort 1, most of the AEs observed in the study were related to the SOC (musculoskeletal and connective tissue disorders); the most common AE was arthralgia. In cohort 2, most of the AE(s) observed in the study were related to the SOC (general disorders and administration site conditions). The most common AEs in the 75 M group were injection site pain and arthralgia. Three events of arthralgia were experienced by two subjects (one subject had two episodes of arthralgia due to OA) and four events of injection site joint pain were experienced by four subjects in the 75 M dose group. One event of hypersensitivity to IMP (joint swelling) was experienced by a subject in the 75 M dose group. Three events of hypersensitivity to IMP (joint swelling) were experienced by three subjects in the 150 M group. All events of joint pain and swelling recovered completely upon symptomatic treatment. Hematology, serum chemistry, serology, urine analyses, and ECG evaluation did not reveal any significant abnormalities.

**Table 5:** Summary of adverse events

	Cohort 1			Cohort 2		
System organ class	25 M (*n* = 10)	50 M (*n* = 10)	P1 (*n* = 10)	75 M (*n* = 10)	150 M (*n* = 10)	P2 (*n* = 10)
Any adverse event	24 (7)	13 (7)	21 (7)	17 (7)	11 (6)	11 (6)
Blood and lymphatic system disorders	0	1 (1)	1 (1)	0	0	0
Endocrine disorders	0	0	0	1 (1)	0	0
Eye disorders	0	1 (1)	0	0	0	0
Gastrointestinal disorders	1 (1)	0	2 (2)	1 (1)	0	3 (3)
General disorders and administration site conditions	2 (1)	4 (2)	0	9 (6)	4 (3)	0
Infections and infestations	3 (3)	1 (1)	4 (3)	0	1 (1)	1 (1)
Injury, poisoning, and procedural complications	0	0	1 (1)	2 (1)	3 (3)	0
Investigations	2 (2)	1 (1)	1 (1)	0	1 (1)	2 (2)
Metabolism and nutrition disorders	4 (3)	0	3 (3)	0	0	1 (1)
Musculoskeletal and connective tissue disorders	6 (3)	3 (3)	5 (4)	4 (2)	2 (2)	3 (2)
Nervous system disorders	1 (1)	0	1 (1)	0	0	0
Renal and urinary disorders	0	1 (1)	0	0	0	0
Reproductive system and breast disorders	1 (1)	0	0	0	0	0
Respiratory, thoracic, and mediastinal disorders	0	0	0	0	0	1 (1)
Skin and subcutaneous tissue disorders	1 (1)	0	1 (1)	0	0	0
Surgical and medical procedures	2 (1)	0	0	0	0	0
Vascular disorders	1 (1)	1 (1)	2 (1)	0	0	0

Values are shown as number of events (number of patients)

25 M, 50 M, 75 M, 150 M = 25, 50, 75, and 150 million cells, respectively

P1, P2 = placebo 1 and placebo 2, respectively

#### Efficacy results

**Fig. 5:**
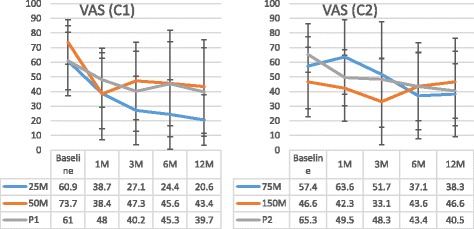
Visual analog scale values. Data are presented as mean ± SD. 1 M, 3 M, 6 M, and 12 M = 1, 3, 6, and 12 months, respectively; *C1* cohort 1, *C2* cohort 2, *M* million cells, *P* placebo, *VAS* visual analog scale

VAS scores decreased over the study period for all the treatment groups except for patients in the 150 M group. The maximum reduction in the VAS score was seen in the 25 M group at 12 months compared to the other groups of patients (40.3 ± 17.3, 30.3 ± 31.0, and 21.3 ± 28.3 cm in 25 M, 50 M, and P1, respectively; *P* = 0.3833). VAS decreased by 67.4% in the 25 M group compared to 41.4% and 36.0% in the 50 M group and P1, respectively (*P* = 0.0587) (Fig. 5).

**Fig. 6:**
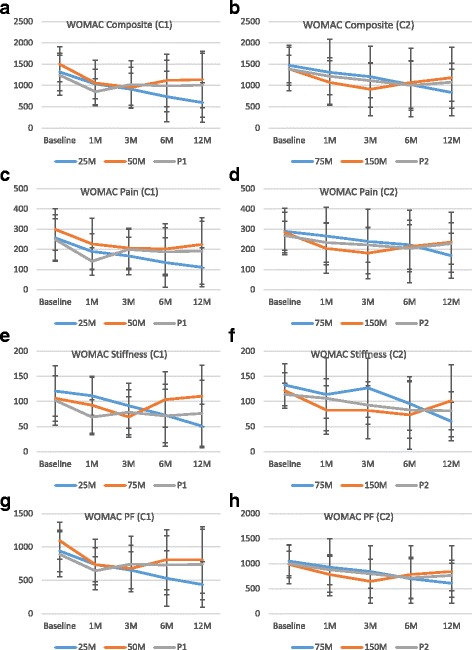
WOMAC results. WOMAC **a, b** composite, **c, d** pain, **e, f** stiffness, and **g, h** physical function (*PF*) results are shown for cohorts 1 (**a**, **c**, **e** and **g**) and 2 (**b**, **d**, **f**, and **h**). Data are presented as mean ± SD. 1 M, 3 M, 6 M, and 12 M = 1, 3, 6, and 12 months, respectively; *C1* cohort 1, *C2* cohort 2, *M* million cells, *P* placebo, *WOMAC* Western Ontario and McMaster Universities

The WOMAC composite index score decreased over the study period for all the treatment groups. The maximum reduction in the WOMAC composite score was seen in the 25 M group at 12 months compared to the other groups (717.8 ± 503.8, 359.9 ± 786.4, and 233.8 ± 641.9 in the 25 M, 50 M, and P1 groups, respectively; *P* = 0.2651). The WOMAC composite index decreased by an mean of 64.8% in the 25 M patients compared to 14.4% and 49.3% in the 50 M and P1, respectively (*P* = 0.1793). A similar trend was observed in all the WOMAC subscores (WOMAC pain reduced by 145.1 ± 105.2, 74.1 ± 167.2, and 57.4 ± 151.3 (*P* = 0.3484); WOMAC stiffness reduced by 69.6 ± 44.7, 4.5 ± 87.2, and 25.8 ± 53.4 (*P* = 0.0324); and WOMAC physical function reduced by 503.1 ± 375.1, 290.3 ± 559.2, and 150.6 ± 457.8 (*P* = 0.2939) in the 25 M, 50 M, and P1 groups, respectively) (Fig. 6).

**Fig. 7:**
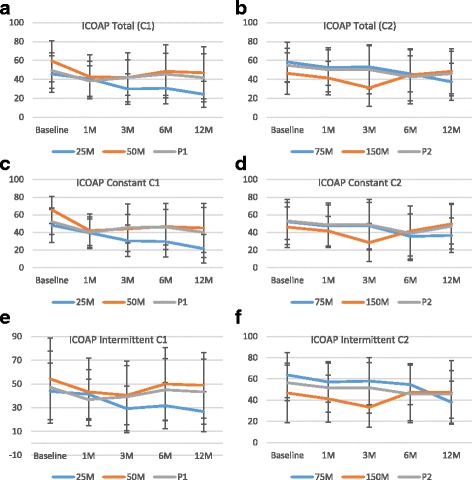
ICOAP results. ICOAP **a, b** total, **c, d** constant pain, and **e, f** intermittent pain results are shown for cohorts 1 (**a**, **c**, and **e**) and 2 (**b**, **d**, and **f**). Data are presented as mean ± SD. 1 M, 3 M, 6 M, and 12 M = 1, 3, 6, and 12 months, respectively; *C1* cohort 1, *C2* cohort 2, *ICOAP* intermittent and constant osteoarthritis pain, *M* million cells, *P* placebo

ICOAP total scores decreased over the study period for all the treatment groups, except the 150 M group. The maximum reduction in the ICOAP total score was seen in the 25 M group at 12 months (21.4 ± 21.2, 12.3 ± 27.4, and 7.5 ± 27.1 in the 25 M, 50 M, and P1 groups, respectively; *P* = 0.5271) (Fig. 7). ICOAP total decreased by 34.6% in the 25 M group compared to 29.0% and 22.2% in the 50 M and P1 groups, respectively (*P* = 0.3844). A similar trend was seen in ICOAP subscores (constant pain reduced by 26.5 ± 25.3, 20.5 ± 30.2, and 12 ± 31.8 (*P* = 0.6140); intermittent pain reduced by 17.1 ± 28.4, 5.4 ± 33.1, and 3.8 ± 26.3 (*P* = 0.6215) in the 25 M, 50 M, and P1 groups, respectively).

Thus, overall the patients in the 25 M group consistently showed pain reduction in all subjective parameters measured in the study. Due to the small sample size, none of the efficacy parameters were statistically significant as the study was not powered for establishing efficacy. There were no clinically meaningful changes in the X-ray parameters at follow-up visits compared to baseline (data not presented). In the MRI evaluation, overall, there was no perceptible change in WORMS score including cartilage signal and morphology from baseline to follow-up visits in any of the groups of patients (Table 6).

**Table 6:** WORMS scoring of MRI of the knee at each visit

WORMS	25 M	50 M	P1	*P* value	75 M	150 M	P2	*P* value
Baseline	67.0 (19.8)	78.8 (40.9)	76.5 (23.5)	–	71.3 (21.4)	62.0 (17.9)	70.8 (14.7)	–
6 months	67.5 (20.5)	77.9 (41.2)	74.9 (22.4)	0.5521	71.4 (20.9)	62.0 (17.7)	69.9 (14.3)	0.7360
12 months	66.1 (19.2)	78.0 (41.1)	74.9 (22.5)	0.5310	67.0 (20.9)	60.6 (15.7)	72.3 (15.2)	0.0609

Values are shown as mean (SD)

The range of WORMS score used in this study was 0–314

25 M, 50 M, 75 M, 150 M = 25, 50, 75, 150 million cells, respectively

P1, P2 = Placebo 1 and 2, respectively

MRI magnetic resonance imaging, WORMS whole-organ magnetic resonance imaging score

## Discussion

The propensity of MSCs to differentiate into chondrocytes in vitro [29] and their ability to repair articular cartilage has been shown in various preclinical models of OA [30–32]. In several studies, MSCs were prepared and injected with sodium hyaluronan to increase the engraftment and chondrogenic activity [30, 33]. In the present study, the efficacy of Stempeucel® was evaluated in a well-validated animal model of OA that was induced by MIA injection into the knee joints. Both low and high doses of Stempeucel® + HA treatment showed significant improvement in the pain threshold from week 2 onwards when compared to animals treated only with HA; treatment with only HA provided a short-term benefit on pain reduction, which corroborates with an earlier publication [34]. We did not observe a significant difference between the two Stempeucel® treatment groups of animals (low and high dose) on pain reduction. However, it is important to note that the pain reduction in the high-dose animals continued to improve until the end of the study (12 weeks). Although the exact mechanism of action of MSCs on pain reduction is not known, anti-inflammatory activity has been attributed to this effect. To date, some studies have demonstrated the role of MSCs on OA-induced pain behavior [35–37]. Van Buul et al. reported improvement of weight-bearing joints of the affected limb after intra-articular application of both rat and human BMMSCs in MIA-induced OA rats [37]. However, unlike the results presented in this study, the authors did not observe cartilage regeneration. Furthermore, in several animal studies, it has been shown that the increased levels of pro-inflammatory cytokines might have contributed to pain increase. Intra-articularly administered MSCs probably play an important role in attenuating the inflammation-induced pain by secreting a wide range of anti-inflammatory cytokines and analgesic peptides [38], and Stempeucel® might have also contributed to pain reduction through a similar mechanism.

We also demonstrated that the pooled BMMSC population are efficient in differentiating into chondrocytes in vitro, and secrete a significant amount of sGAG (Fig. 2c). When these cells were administered intra-articularly into OA-affected joints, we observed a progressive increase in proteoglycan staining. The improvement in cartilage repair was observed both macroscopically and microscopically. The sGAG intensity data revealed that the total proteoglycan content was significantly higher in both the cell + HA treated groups compared to animals treated only with HA. One of the short comings of the preclinical results is that we did not determine the therapeutic effect of BMMSCs without HA. However, based on the published data it appears that administration of MSCs in combination with HA provided better therapeutic benefit than either HA or MSC treatment alone in an experimental animal model of OA [30]. The concomitant reduction in MIA-induced pain followed by an increase in cartilage regeneration observed in this study suggests that human bioactive factors synthesized by BMMSCs may be responsible for both the reduction in inflammation and promotion of endogenous cartilage regeneration via a paracrine mechanism [12].

This clinical study met its predefined endpoint of safety of intra-articular administration of Stempeucel® in osteoarthritis of the knee joint. Adverse events were predominantly local pain and swelling, particularly seen in patients randomized to the higher dose groups (75 M and 150 M) and they resolved completely upon symptomatic treatment. There was no evidence of ectopic tissue or tumor formation locally at 1-year follow-up. Hematological, biochemical, and serological parameters were comparable in both the cell and placebo arm in all groups of patients. Limited joint space, higher dose, and volume of injection (6 ml) may be the reason for increased joint swelling and pain seen in cohort 2 (75 M and 150 M). Furthermore, it can be assumed that a proportion of the cells injected into the joint space have not survived and this phenomenon was more pronounced with higher cell doses. Probably, such non-viable cells produce an inflammatory reaction causing pain and swelling, as reported earlier [39]. The frequency of these complications was similar to a report from another study using culture-expanded bone marrow-derived MSCs [40]. In another study using allogeneic non-HLA matched BMMSCs in two different doses (50 and 150 million cells) which were pre-mixed with hyaluronic acid (5 ml) and administered in partial medial meniscectomy patients [10], the adverse events were similar to those seen in our study, with the most frequently reported AE by system organ class being musculoskeletal and connective tissue disorders [10]; however, the adverse events did not differ between the two doses tested. Recently, Vega et al. have conducted a study using IA injection of allogeneic BMMSCs (40 million cells suspended in 8 ml of Ringer-Lactate) in OA of the knee joint [11]. Post-implantation pain was observed in 53% to 60% of patients in both the experimental and control groups. The pain responded to analgesics and improved within 1 to 6 days. Hence, pain and local swelling may be the most common post-injection complication in patients after IA injection of MSCs which responds within a few days of symptomatic treatment.

One of the most important factors influencing the clinical outcome of a study is to determine the optimal treatment dose. In this study, patients in the low-dose group (25 million cells) showed improved outcomes in the pain measurement scores, whereas those in the higher dose groups did not. The VAS and WOMAC composite index scores decreased by 64% and 64.4% in the 25-million-cell arm as compared to 36% and 49.3% in the active controls with HA, respectively, at 12 months follow-up. In a proof of concept study, three doses of autologous adipose tissue-derived MSCs (AD-MSCs) were used: 10 million, 50 million, and 100 million cells. The WOMAC score improved at 6 months follow-up in the high-dose group [14]. In another study using allogeneic BMMSCs at a dose of 40 million cells, improvement in pain, disability, quality of life, and cartilage quality by MRI was noted in the cell-treated group [11]. Several reasons are hypothesized for this effect in the low-dose group of patients as observed in this study. Firstly, a dose of 25 M cells may be optimum with the volume of hyaluronic acid (2 ml) used in the study as a supporting matrix. Secondly, the 25-million-cell dose maybe optimal for the limited IA space in the knee joint. Thirdly, doses higher than 25 million might cause cell aggregation due to a high cell concentration or insufficient space in the knee joint and subsequently cause cell death. Fourthly, the 25-million-cell dose may be lying in the upper range of the efficacy dose since numerous studies reports that doses in the range of 10 to 25 million BMSCs may be efficacious in OA of the knee joint [15, 41–45]. Finally, higher doses of MSCs may activate the MSCs to function as an M1-type cell with a pro-inflammatory response [46], whereas the 25 M dose may be the optimal concentration of cells which gives rise to an M2-type MSC with an anti-inflammatory/immunosuppressive response. Hence, which cell dose will lead to the best outcome cannot be determined until a series of dose-finding studies are carried out.

Various studies are ongoing to determine the optimal tissue source of MSCs for therapeutic repair of the cartilage tissue. The combination of MSCs with scaffolds, growth factors, platelet-rich plasma (PRP), and genetic modification have also been studied. It is not clear which source of stem cells, or a combination product, will be the best for the disease condition. Studies have shown that adipose tissue-derived stem cells are both safe and efficacious [13, 14, 47–49], whereas other studies have shown that bone marrow-derived cells are equally efficacious [10, 11, 50–52]. A current focus for knee cartilage repair is to use scaffolds that provide a three-dimensional environment for guiding and supporting the cells for cartilage repair. An advantage for using a scaffold is containment of the implanted cells on the lesion, and these biomaterials may act as barriers for fibroblast invasion of the graft [53, 54]. Koh et al. have used PRP as a scaffold as it acts as an MSC accelerator for clinical chondrogenesis, is non-immunogeneic and bioabsorbable, and can be easily prepared preoperatively [13]. In another study, fibrin glue has been used as a scaffold in MSC implantation to induce improved cell survival, proliferation, gene expression, differentiation, and matrix synthesis leading to repair of the cartilage lesion [55]. Cartistem® (MEDIPOST Co. Ltd., South Korea) is a combination product of human umbilical cord blood-derived mesenchymal stem cells and hyaluronic acid [56]. This acts as a biodegradable matrix in MSC implantation as it facilitates the migration and adherence of cells to the damaged cartilage, leading to better healing of the damaged lesion. Hence, more studies are required to pinpoint the best source of stem cells and the scaffold to be used to demonstrate both safety and efficacy.

The method of delivery of cells—either by direct intra-articular injection or by open arthroscopy injection—into the joint cavity is also important and may be one of the factors for deriving efficacy. In one of the initial studies, Wakitani et al. transplanted cells of bone marrow embedded in collagen gel into the articular cartilage defect at the time of high tibial osteotomy [43]. Cartistem®, a combination product approved by the Korean FDA, has been applied to the damaged area through arthroscopy after conducting a microfracture [57]. These open surgical methods have their disadvantages such as pain, longer hospital stay, and higher cost. Minimally invasive techniques such as intra-articular injection have been adopted by different groups [14, 15, 41, 45, 50]. IA injection is patient-friendly in terms of being less invasive, with reduced hospital stay, and are likely to reach a larger patient population as it can be performed in peripheral hospitals. Ultrasound guidance of knee injections could be a better option to more precisely deliver the cells intra-articularly. Berkoff et al. have reported that ultrasound guidance of knee injections resulted in better IA accuracy of needle placement than anatomical guidance (95.8% versus 77.8%; *P* < 0.001) [58]. This enhanced injection accuracy achieved with ultrasound needle guidance directly improves patient-related clinical outcomes. However, in developing countries, ultrasound-guided intra-articular injection may be a challenge due to limited access to the instrument.

The present study, though it has shown good subjective improvement in pain and functional scores, did not demonstrate improvement in cartilage signal and morphology by MRI. We have used the WORMS scoring system, which is a semiquantitative MRI system for evaluating structural change in knee OA. WORMS scoring has been extensively studied for the prevalence and severity of cartilage loss, bone marrow lesions, and meniscal damage [59, 60], and has seldom been studied for cartilage regeneration. Koh et al. studied the effect of adipose-derived MSCs with PRP in OA of the knee joint and found that WORMS score significantly improved from 60.0 points to 48.3 points and cartilage subscore improved from 28.3 points to 21.7 points at 24 months follow-up (*P* < 0.001) as compared to baseline [13]. However, in our study, the cartilage subscore did not demonstrate any significant worsening or improvement of the cartilage in any of the subgroups. The reason for the WORMS score differences between these two studies could be due to several reasons: the type of MSCs used are different, better complementarity between adipose-derived MSC and PRP, or the length of follow-up time (24 months vs. 12 months) after cell administration. The limited number of patients used in this study for MRI analysis might have contributed as well. Regardless of these differences, Stempeucel® administration in the preclinical model clearly suggested the ability of these cells in combination with HA to trigger adequate proteoglycan synthesis for cartilage repair. Future clinical trials of Stempeucel® in OA patients should consider using guided delivery of cells in and around the lesion site or by arthroscopy, followed by MRI measurements using delayed gadolinium-enhanced MRI of cartilage (dGEMRIC) or T2 mapping to perform compositional (sGAG) analysis of the cartilage before and after cell administration. One of the limitations of this study was unblinding of the trial after 6 months follow-up, particularly given that the subjective measurements of clinical data (VAS, WOMAC, and ICOAP) were the secondary endpoints.

## Conclusions

This clinical study satisfied the primary endpoints of safety of Stempeucel® administration in OA patients at all four doses tested. In addition, a trend towards pain reduction at the lowest cell dose of 25 M was observed by VAS, WOMAC, and ICOAP pain scoring criteria, but this was not statistically significant when compared to placebo. Analysis of the remaining secondary endpoints did not reveal therapeutic efficacy which could be attributed to the low number of patients enrolled in the study. The fact that a pooled population of allogeneic BMMSCs could elicit pain reduction and cartilage regeneration in a preclinical model of OA coupled with the safety profile observed in human patients with a positive trend in pain reduction (in one of the cell doses tested) warrants further study in a large number of patients to investigate the therapeutic role of Stempeucel® in cartilage regeneration in OA patients.

## Abbreviations

AE: Adverse event
BMI: Body mass index
BMMSC: Bone marrow-derived mesenchymal stromal cell
dGEMRIC: Delayed gadolinium-enhanced magnetic resonance imaging of cartilage
DMSO: Dimethyl sulfoxide
GCP: Good Clinical Practice
GMP: Good Manufacturing Practice
HA: Hyaluronic acid
HED: Human equivalent dose
IA: Intraarticular
ICH: International Conference on Harmonization
ICOAP: Intermittent and constant osteoarthritis pain
IMP: Investigational medicinal product
MedDRA: Medical Dictionary for Regulatory Activities
MIA: Mono-iodoacetate
mITT: Modified intention to treat
MRI: Magnetic resonance imaging
MSC: Mesenchymal stromal cell
OA: Osteoarthritis
PAM: Pressure Application Measurement
PT: Preferred term
SAE: Serious adverse event
sGAG: sulfated glycosaminoglycan
SOC: System organ class
VAS: Visual analog scale
WCB: Working cell bank
WOMAC-OA: Western Ontario and McMaster Universities Osteoarthritis
WORMS: Whole-organ magnetic resonance imaging score
